# Overexpression of the orphan nuclear receptor NR2F6 is associated with improved survival across molecular subgroups in endometrial cancer patients

**DOI:** 10.1007/s00432-023-04632-2

**Published:** 2023-03-08

**Authors:** L. Proppe, T. Jagomast, S. Beume, L. Klapper, G. Gitas, F. Köster, S. Perner, A. Rody, J. Ribbat-Idel, L. C. Hanker

**Affiliations:** 1 Department of Gynecology and Obstetrics, University Hospital Schleswig-Holstein, Campus-Lübeck, Ratzeburger Allee 160, 23562 Luebeck, Germany; 2 Department of Pathology, University Medical Center Schleswig-Holstein, Campus-Lübeck, Lübeck, Germany; 3 Department of Gynecology and Obstetrics, University Medical Center Charité Berlin, Berlin, Germany

**Keywords:** Endometrial cancer, NR2F6, Molecular subgroups, Immune checkpoint inhibition

## Abstract

**Introduction:**

NR2F6 (nuclear receptor subfamily 2 group F member 6, also called Ear-2) is known to be an orphan nuclear receptor that has been characterized as an intracellular immune checkpoint in effector T cells and, therefore, may control tumor development and growth. The prognostic impact of NR2F6 in endometrial cancers is evaluated in this study.

**Materials and methods:**

Expression analysis of NR2F6 in 142 endometrial cancer patients was performed by immunohistochemistry of primary paraffin‑embedded tumor samples. Staining intensity of positive tumor cells was automatically assessed semi-quantitatively, and results were correlated with clinicopathological characteristics and survival.

**Results:**

Forty five of 116 evaluable samples (38.8%) showed an overexpression of NR2F6. This leads to an improvement of the overall survival (OS) and progression-free survival (PFS). In NR2F6-positive patients, the estimated mean OS was 156.9 months (95% confidence interval (CI) 143.1–170.7) compared to 106.2 months in NR2F6-negative patients (95% CI 86.2–126.3; *p* = 0.022). The estimated PFS differed by 63 months (152 months (95% CI 135.7–168.4) vs. 88.3 months (95% CI 68.5–108.0), *p* = 0.002). Furthermore, we found significant associations between NR2F6 positivity, MMR status, and PD1 status. A multivariate analysis suggests NR2F6 to be an independent factor influencing the OS (*p* = 0.03).

**Conclusion:**

In this study, we could demonstrate that there is a longer progression-free and overall survival for NR2F6-positive patients with endometrial cancer. We conclude that NR2F6 might play an essential role in endometrial cancers. Further studies are required to validate its prognostic impact.

**Supplementary Information:**

The online version contains supplementary material available at 10.1007/s00432-023-04632-2.

## Introduction

The orphan nuclear receptor NR2F6 (nuclear receptor subfamily 2 group F member 6, also called Ear-2) has been recently shown to be part of the cellular, innate immune system. NR2F6 is member of the COUP-TF (chicken ovalbumin upstream promoter transcription factor) and is a regulatory intranuclear receptor, which binds directly to DNA, including a control function of gene regulation and cell differentiation (Arora et al. 2018; Chang et al. 2019).

In 2018, Klepsch et al. described a correlation of NR2F6 expression and the PD1/PD-L1-signaling pathway. They showed that tissue from *NR2F6*-deficient mice was associated to higher PD1- and PD-L1 expression on CD4- and CD8-positive T cells. Their results suggest NR2F6 to be another potential target in immune checkpoint therapy (Klepsch et al. 2018). Among all gynecologic cancers, endometrial carcinomas have a relatively high tumor mutational burden and are, therefore, promising targets in immune checkpoint therapy, as for example with the PD1-inhibitor Dostarlimab (The Cancer Genome Atlas Research Network und Levine 2013). Immune checkpoint therapy will determine future cancer treatment.

The PD1/PD-L1 signaling pathway plays a crucial role in tumor progression. Tumor cells may be able to inhibit tumor-infiltrating lymphocytes (TILs) via binding the PD1-receptor. Klepsch et al. found evidence that PD1-inhibition and down-regulation of NR2F6 in tumor-infiltrating lymphocytes strongly act in synergistic manners (Klepsch et al. 2018).

Endometrial cancer is the most common gynecologic cancer (Siegel et al. 2020). Due to a rise of the well-known risk factors in the western population, especially the metabolic syndrome, the incidence of endometrial carcinomas is rising (Yang et al. 2013; Arora et al. 2018). Early-stage endometrial carcinomas generally have a good prognosis, while patients with FIGO III and IV endometrial cancer stages do have a very poor 5-year survival prognosis. The recent identification of molecular subgroups has a strong impact on the treatment of endometrial cancers in all tumor stages (Chang et al. 2019; Concin et al. 2021).

The individual immune response is an essential part concerning carcinogenesis and tumor growth. T-cell activation is a crucial strategy to prevent tumorigenesis. CD8-positive T cells may eradicate tumor cells MHC-associated or through cytokine production. CD4-positive T cells are involved as well and operate mainly by differentiation of tumor-infiltrating macrophages (Klepsch et al. 2016). These endogenous immune responses are often actively suppressed by the tumor (Wherry and Kurachi 2015). Applying immune checkpoint therapies, the individual immune cells get enabled to operate against the tumor (Kasherman et al. 2021).

The endogenous mechanism of activation or inactivation of NR2F6 is not yet known. In vitro studies revealed NR2F6 acting as inhibitory receptor, highly expressed on so called “exhausted” T cells, as they might be found in tumor tissue due to a tumor-related unregulated T-cell activation process (Wherry and Kurachi 2015; Klepsch et al. 2021). Furthermore, NR2F6 seems to modulate anti-inflammatory signals between T cells, which may ameliorate the individual anti-tumor response. However, the data from animal experiments published by Klepsch et al. showed generally a better survival in *NR2F6*-deficient mice compared to *NR2F6*-proficient mice, suggesting that these mice benefit from the *NR2F6* deficiency in terms of tumor growth and immune response concerning cancer cells (Klepsch et al. 2021).

An increased amount of tumor-infiltrating lymphocytes (TIL) has been shown to be associated with a better survival in cancer patients (Loi et al. 2019). Nevertheless, the individual role of TIL, the immune system in general and the tumor cells are still subject of current research. Several studies have shown that NR2F6 overexpression may lead to a certain chemotherapy resistance of different carcinomas (Klepsch et al. 2021; Loi et al. 2019). Recent research revealed that many oncologic associations are multifactorial and, often, synergistic effects of different pathways need to be considered.

The aim of this study is the exploration of the orphan nuclear receptor NR2F6 aiming a contribution to future, immune-guided cancer treatment.

## Materials and methods

**Fig. 1:**
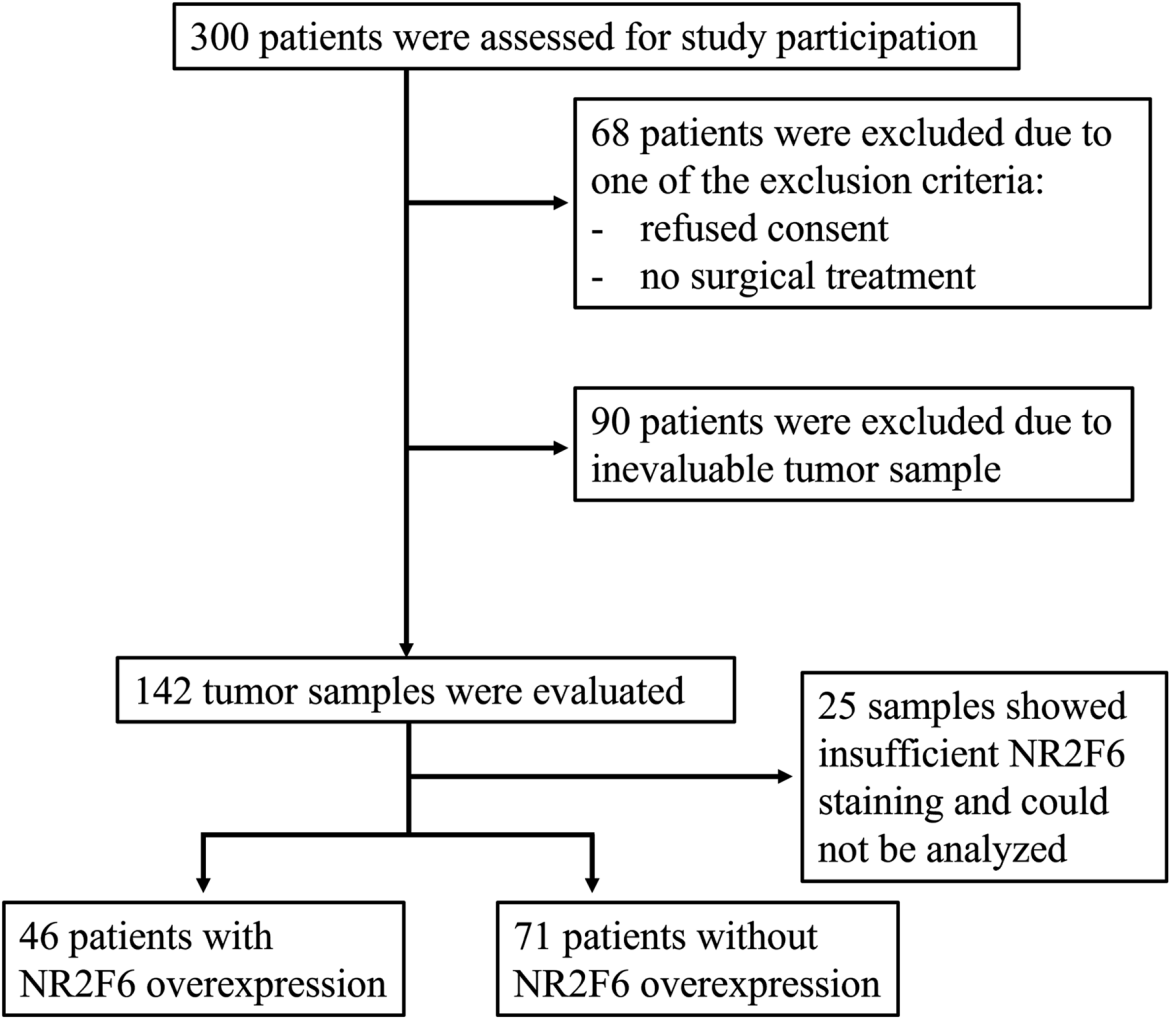
Consort diagram

Patients treated surgically for primary endometrial cancer in the department of gynecology at the University Hospital Lübeck, Germany in the years 2006–2018 were included retrospectively. The study was performed in compliance with the Helsinki Declaration and approved by the ethics committee of the University of Lübeck (19-082A). Exclusion criteria were no surgical treatment, the absence of patient consent, and the absence of adequate tumor tissue. One hundred and forty two patients finally met the inclusion criteria (Fig. 1).

Medical history, details of surgery, histology, tumor stage, and postoperative data were reviewed. A questionnaire was used to collect data concerning the disease-free survival. These clinical data were correlated with results from immunohistochemical staining.

To perform immunohistochemical staining, paraffin-embedded tumor samples were first arranged into slides and subsequently stained with hematoxylin and eosin. The slides were then evaluated by an experienced pathologist (JR), the region of tumor cells was marked, and these samples then arranged to tissue microarrays (TMAs). Each TMA consisted of up to 60 1 mm^2^ samples, thereof 6 normal tissue samples and up to 54 tumor tissue samples.

The NR2F6 antibody (rabbit polyclonal, 1:50, ab137496, Abcam, Cambridge, UK) was used to evaluate the NR2F6 status of each tumor sample. Furthermore, the Estrogen receptor und Progesterone receptor were analyzed using anti-Estrogen Receptor (SP1) Rabbit Monoclonal and anti-Progesterone Receptor (1E2) Rabbit Monoclonal (Ventana, Roche, Basel, Switzerland). For each staining, the IView DAB Detection Kit was used on a Ventana Bench Mark automated staining system (Roche, Basel, Switzerland).

The stained TMAs were then scanned using the Ventana iScan HT scanner (Ventana Tucson, AZ, USA). The samples were computerized and semi-automatically analyzed using the Definiens Tissue Studio software (Definiens Developer XD 2.0, Definiens AG, Munich, Germany). NR2F6 overexpression is defined with respect to the mean of the NR2F6 staining intensity.

Statistical analyses were performed using IBM SPSS Statistics for Windows (SPSS Statistics, v 27, IBM Corp., Armonk, NY, USA). The immunohistochemical staining intensity has been correlated to clinicopathological characteristics and survival data. We hypothesized that NR2F6 overexpression correlates to a worse survival of the patients. We used the ${\chi }^{2}$-test or Fisher’s exact test to compare frequencies among groups. To analyze survival data, Kaplan–Meier curves and the log-rank test were applied. For multivariate analyses, the Cox regression method was used. A *p*-value below or equal to 0.05 was considered significant.

## Results

Mean age of all patients was 67.5 ± 12.2 years, the mean BMI amounted to 30.1 ± 8.4 kg/m^2^ (SD). The majority of the patients was classified in tumor stages FIGO I or II. 62.7% of the patients received a systematic lymphadenectomy, but only 14 patients (9.9%) finally had lymph node metastases. According to the pathologic reports, 84.5% of the tumors had an endometrioid histology and 60.6% of the patients were surgically treated by laparoscopy.

Baseline characteristics of all patients are shown in Table 1.

**Table 1:** Baseline characteristics and clinical data of patients (*n* = 142)

Patient characteristics		*n* (%)
Age (years)	> 60	104 (72.5%)
	≤ 60	38 (26.8%)
FIGO	I–II	107 (75.4%)
	III–IV	33 (23.2%)
BMI (kg/m^2^)	> 30	54 (38.0%)
	≤ 30	78 (54.9%)
Surgery technique	Laparoscopy	86 (60.6%)
	Laparotomy	51 (35.9%)
Lymph node metastases	yes	14 (9.9%)
	No	69 (48.6%)
Grading	G1	72 (50.7%)
	G2 or G3	67 (47.2%)
p53 status	p53 aberrant	57 (40.1%)
	p53 wild type	75 (52.8%)
MMR status	MMR deficient	56 (39.4%)
	MMR proficient	77 (54.2%)
Ki -67 status	Ki-67 ≥ 25%	33 (23.2%)
	Ki-67 < 25%	98 (69.0%)
Histopathology	Endometrioid carcinoma	120 (84.5%)
	Others (e.g., serous or clear cell carcinomas)	21 (14.8%)

The clinical data were then analyzed dependent on the NR2F6 status. Failed staining lead to finally 117 tumor samples that could be evaluated. There was no significant difference between the NR2F6-positive and negative groups concerning the age, BMI, lymph node status, Ki-67 and FIGO stage. The p53-status, Grading and the histology were also not associated to NR2F6 expression. Only MMR status was associated to the NR2F6 expression as there were more patients with a NR2F6-negative tumor and MMR proficiency compared to NR2F6-positive tumors, as depicted in Table 2.

**Table 2:** Clinical data dependent on the NR2F6 status

Characteristic		Total (*n* = 117)	NR2F6 positive (*n* = 46)	NR2F6 negative (*n* = 71)	*p*-value
Age (average, years)		67.5 ± 12.2	63.9 ± 12.0	68.0 ± 12.6	n.s.
FIGO I–II		88 (75.2%)	39 (33.3%)	49 (41.9%)	n.s.
FIGO III–IV		27 (23.1.2%)	5 (4.3%)	22 (18.8%)	0.02
BMI (average, kg/m^2^)		30.1 ± 8.4	29.8 ± 7.5	30.5 ± 9.5	n.s.
Histology	Endometrioid	104 (88.9%)	42 (35.9%)	62 (52.9%)	n.s.
	Other	12 (10.3%)	4 (3.4%)	8 (6.8%)	n.s.
p53 status	Aberrant	48 (41%)	15 (12.8%)	33 (28.2%)	n.s.
	Wild type	68 (58.1%)	31 (26.5%)	37 (31.6%)	n.s.
MMR status	deficient	71 (60.7%)	20 (17.1%)	51 (43.6%)	0.003
	Proficient	45 (38.5%)	25 (21.4%)	20 (17.1%)	n.s.
Grading (G)	G1 or G2	93 (79.5%)	38 (32.5%)	55 (47%)	n.s.
	G3	24 (20.5%)	8 (6.8%)	16 (13.7%)	n.s

**Fig. 2:**
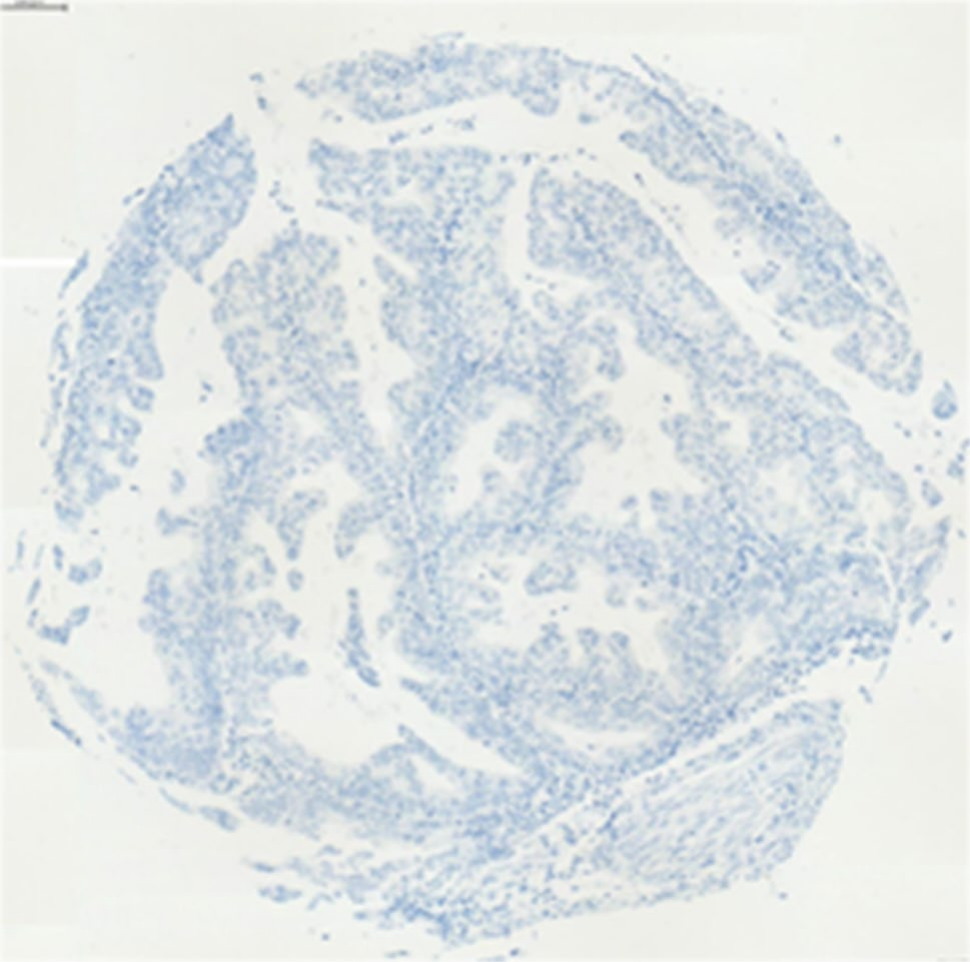
Negative NR2F6 antibody staining

**Fig. 3:**
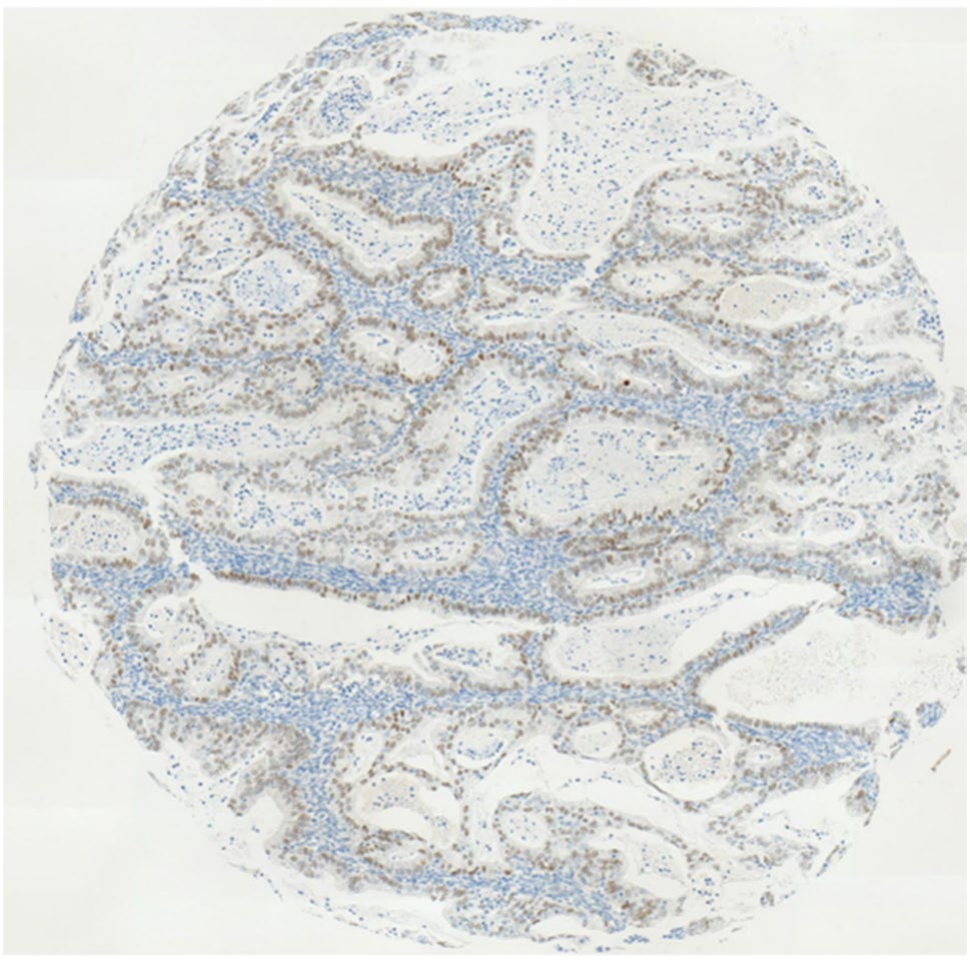
Moderate staining intensity

**Fig. 4:**
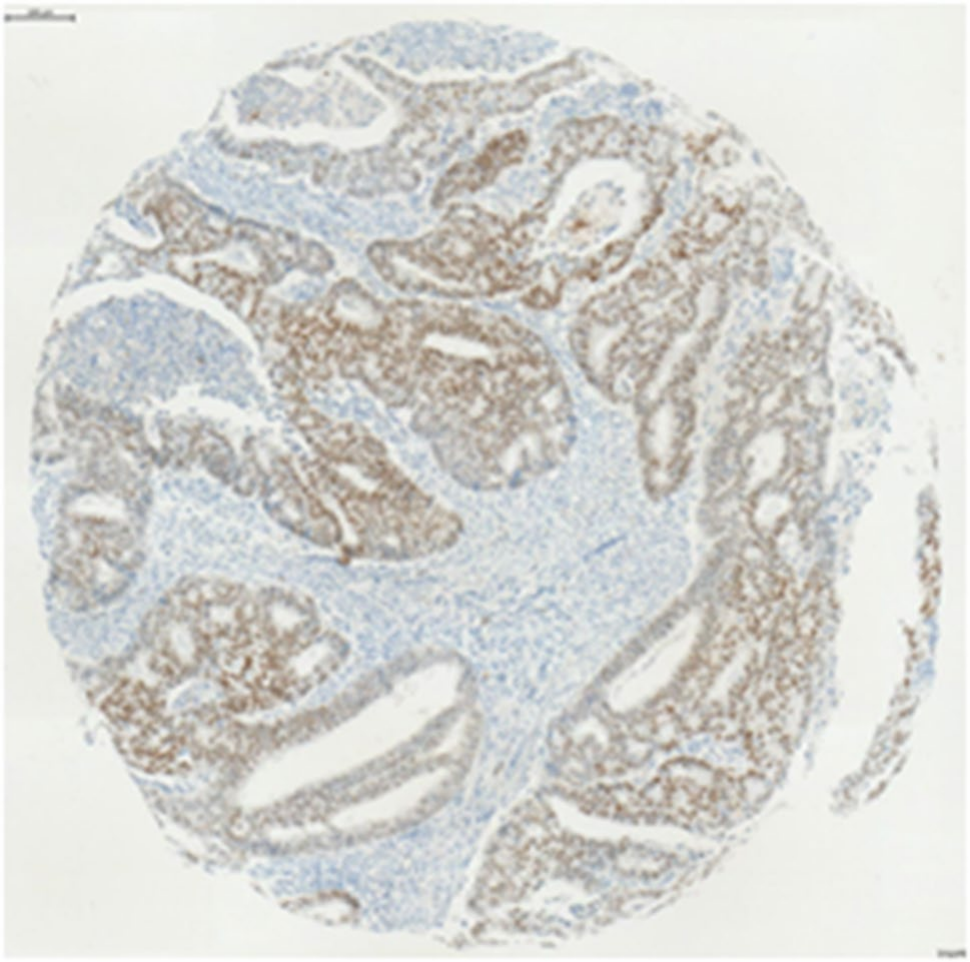
Intense staining, defined as NR2F6 overexpression

Immunohistochemical staining showed a wide variation of staining intensity. As shown in Figs. 2, 3 and 4, the samples varied from completely negative for NR2F6 staining (Fig. 2) to a highly positive status (Fig. 4).

**Fig. 5:**
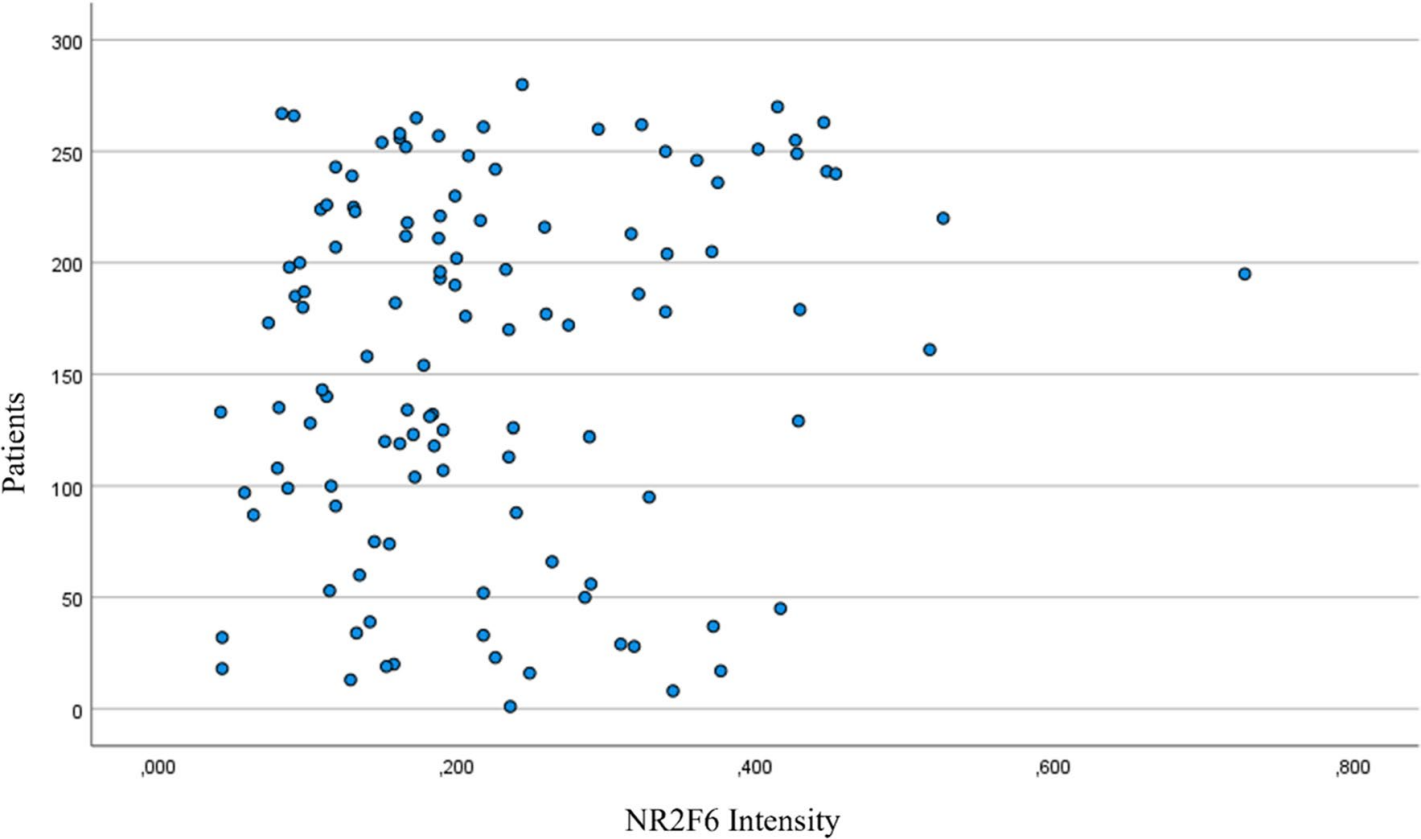
Distribution of expression levels of NR2F6 staining intensity

To analyze NR2F6 expression, the staining intensity was documented for all samples. Regarding gynecological cancers, there are no valid data concerning the evaluation or meaning of NR2F6 staining. Consequently, there were no validated thresholds available. We defined the mean of staining intensity to be the threshold for negativity and positivity. See distribution of NR2F6 staining intensity in Fig. 5.

We compared different subgroups according to immunohistochemical tumor properties using the ${\chi }^{2}$-test. NR2F6 overexpression occurs significantly more often in tumors with MMR-deficiency (*p* = 0.003). Moreover, PD1-negative tumors are more often NR2F6 negative than NR2F6 positive (*p* = 0.02).

**Fig. 6:**
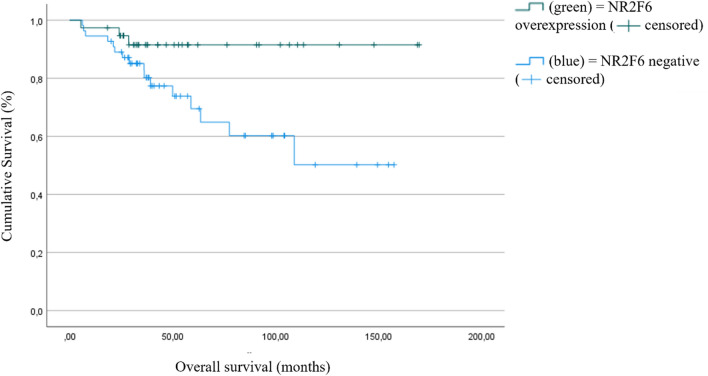
Overall survival (OS) as a function of NR2F6 expression (*p* = 0.022, high NR2F6 expression *n* = 38, low NR2F6 expression *n* = 55)

To evaluate differences in the overall and progression-free survival between NR2F6-negative and NR2F6-positive tumors, the Kaplan–Meier and log-rank tests were used. Patients with NR2F6 expression had a significantly longer overall survival than those without NR2F6 expression. The estimated mean OS was 156.9 months (95% confidence interval (CI) 143.1–170.7) compared to 106.2 months in NR2F6-negative patients (95% CI 86.2–126.3; *p* = 0.022, Fig. 6).

**Fig. 7:**
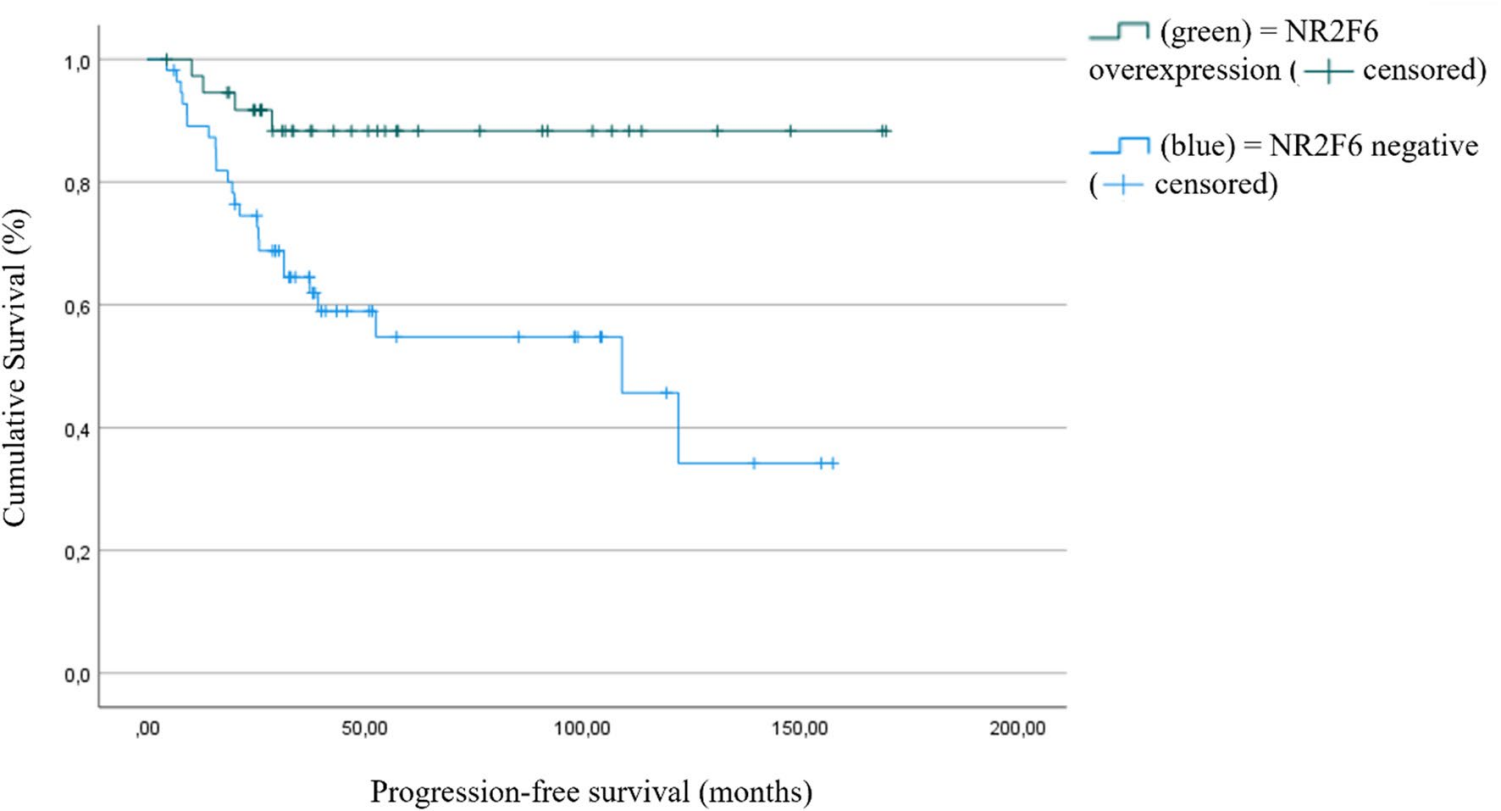
Progression-free survival (PFS) as a function of NR2F6 expression (*p* = 0.002, high NR2F6 expression *n* = 38, low NR2F6 expression *n* = 56)

Furthermore, the progression-free survival is significantly longer in patients with NR2F6-expressive tumors than in those without NR2F6 expression. The estimated progression-free survival differed by 63 months (152 months (95% CI 135.7–168.4) vs. 88.3 months (95% CI 68.5–108.0), *p* = 0.002, Fig. 7). Further results using the median staining intensity as the threshold are shown in the supplemental information.

We performed a subgroup analysis in order to describe the impact of NR2F6 in the most common molecular subgroups of endometrial cancer, i.d. MMRd and p53. Concerning the survival data of p53-positive tumors depending on NR2F6 expression, there were very few patients showing a p53-aberrant tumor and NR2F6 expression. In general, patients with both a p53 wild type and NR2F6 expression showed the longest overall and progression-free survival in this subgroup analysis (*p* = 0.05 (OS) and 0.025 (PFS), respectively).

**Fig. 8:**
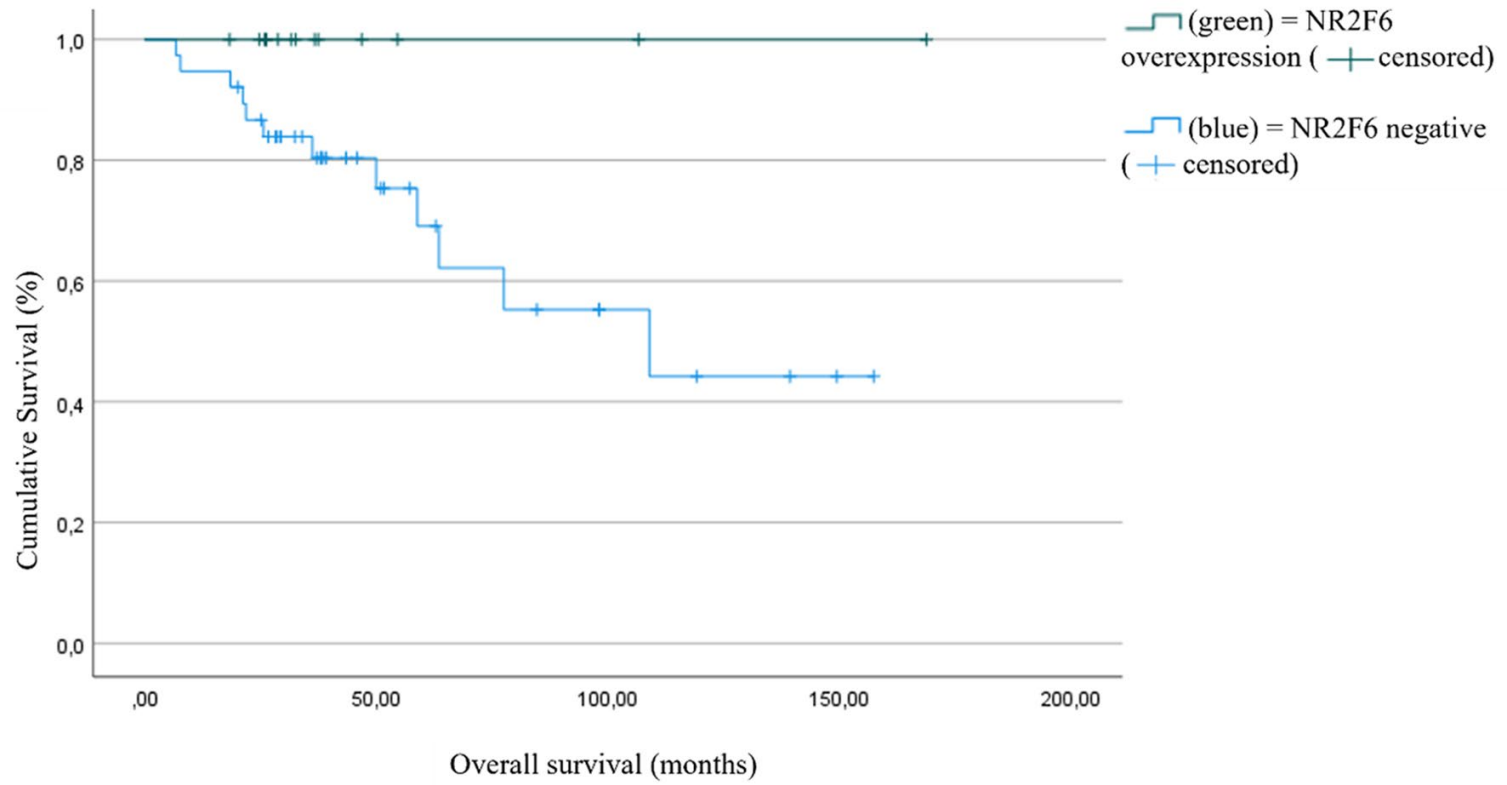
Overall survival (OS) in MMR-deficient patients as a function of NR2F6 expression (*p* = 0.05, high NR2F6 expression *n* = 14, low NR2F6 expression *n* = 38)

For the subgroup of MMR-deficient patients, there is a significant difference concerning the overall and progression-free survival (*p* = 0.05 and *p* = 0.03, respectively). The statistical analysis of the subgroup analysis is constrained by the small sample size. Nevertheless, NR2F6 overexpression shows a better survival in MMR-deficient patients (Fig. 8).

The multivariate Cox regression could be performed in 92 and 93 tumor samples, as their information concerning overall or progression-free survival status and the examined factors was complete. The analysis suggests NR2F6 expression as an independent prognostic marker concerning the overall survival (*p* = 0.03, see Table 3). NR2F6 status and patient’s age were found to be statistically significant (*p* = 0.03 and *p* = 0.02, respectively).

**Table 3:** Results of the multivariate Cox regression analysis concerning the overall survival

Tumor’s property	*n* = 92	Hazard ratio	95% confidence interval		*p*-value
NR2F6 status (neg. vs. pos.)	54 vs. 38	4.116	1.124	15.068	**0.03**
Age (< 60 vs. ≥ 60 years)	34 vs. 58	0.218	0.062	0.766	**0.02**
FIGO stage (I or II vs. III or IV)	72 vs. 20	0.425	0.160	1.128	0.09
MMR (deficient vs. proficient)	52 vs. 40	1.048	0.344	3.198	0.94
p53 (wild type vs. aberrant)	56 vs. 36	0.845	0.32	2.233	0.73
Grading (1 or 2 vs. 3)	73 vs. 19	0.652	0.186	2.284	0.5

Significant values in bold. A *p*-value below or equal to 0.05 is considered to be significant

The multivariate Cox regression analysis shows likewise that the progression-free survival is dependent on the NR2F6 status (*p* = 0.02, Table 4).

**Table 4:** Results of the multivariate Cox regression analysis concerning the progression-free survival

Tumor’s property	*n* = 93	Hazard ratio	95% confidence interval		*p*-value
NR2F6 status (neg. vs. pos.)	55 vs. 38	3.767	1.248	11.374	**0.02**
Age (< 60 vs. ≥ 60 years)	33 vs. 60	0.352	0.131	0.947	0.04
FIGO stage (I or II vs. III or IV)	74 vs. 19	0.955	0.38	2.401	0.92
MMR (deficient vs. proficient)	53 vs. 40	2.267	0.863	5.953	0.1
p53 (wild type vs. aberrant)	56 vs. 37	0.641	0.297	1.382	0.3
Grading (1 or 2 vs. 3)	73 vs. 20	0.484	0.194	1.207	0.12

Significant values in bold. A *p*-value below or equal to 0.05 is considered to be significant

## Discussion

This is the first study investigating the role of the orphan nuclear receptor NR2F6 in endometrial cancer tissue. The results suggest that NR2F6 may play an important role in the cell cycle and carcinogenesis of endometrial cancers.

Our results show a high correlation of NR2F6 expression and survival. These are new insights into survival analyses revealing the prognostic impact of NR2F6 across the most common molecular subgroups in endometrial cancer patients. Unexpectedly, our survival results are contrary compared to the rare data concerning oropharyngeal, ovarian, and cervical cancers (Li et al. 2019; Niu et al. 2016; Klapper et al. 2020). These studies showed that NR2F6 overexpression may be associated to a poorer survival. Additionally, cell culture analyses suggest an association of NR2F6 positivity and chemoresistancy (Zhang et al. 2020; Li et al. 2019). Nevertheless, the endogenous ligand(s) of NR2F6 is/are still unknown. Some authors suggest that an overexpression of NR2F6 reduces the cytokine production and promotes tumor growth this way (Klepsch et al. 2016). The role of NR2F6 in the endometrial adenocarcinoma may be different compared to squamous cell carcinomas as of the oropharynx or cervix uteri. Given that NR2F6 is a transcriptional factor, it may influence the microenvironment of a tumor in multiple ways. In hematopoetic cells, it has been shown that the transcriptional factor NR2F6 may inhibit some cell differentiation and enhance others at the same time (Ichim et al. 2018). As Ichim et al. conclude, NR2F6 might inhibit the growth of certain cells in specific differentiation stages.

The survival analyses in this study show that an NR2F6 overexpression is associated to a better disease-free and overall survival in endometrial cancer patients, respectively. We assume NR2F6 being part of cell signaling pathways which may cause differences of tumor growth in general and, therefore, lead to different survival times.

The clinical characteristics of our cohort are in line with other clinical studies (Yang et al. 2013; Creutzberg et al. 2000; Candido et al. 2019; Volpi et al. 2019). 76.1% of all patients received a systematic lymph node dissection, but only 14 patients finally had lymph node metastases.

We aimed to analyze if the NR2F6 expression may serve as an independent prognostic marker. Indeed, although NR2F6 expression was inversely associated to MMRd, it did not correlate with p53. Its prognostic impact was also confirmed independently from molecular subgroups like MMRd and p53 in a multivariate COX regression analysis. In this study, we mainly included endometrioid endometrial cancer and we did not specify the POLE status. Our results suggest, that NR2F6 may influence independently from molecular subgroups the prognosis of endometrial cancer patients. However, the interpretation of the subgroups is limited due to the small sample size. Additionally, it is an important issue to evaluate and interpret the immunohistochemical results. There is still lack of evidence due to missing studies and guidelines with distinct thresholds to evaluate staining intensity. Deviating from literature reports, we identified relatively high amounts of patients with p53-aberrant or MMRd tumors. It might reflect changes due to long storage and difficulties of the immunohistochemical staining interpretation as it is known as the inter-observer variability in many immunohistochemical analyses. Both should be further investigated. Additionally, as already mentioned, NR2F6-positive peritumoral lymphocytes which may significantly influence tumor’s microenvironment were not analyzed in this study. The impact of peritumoral lymphocytes may be an explanation for varying (survival) results in different studies. A validation of all immunohistochemical parameters is urgently needed.

To determine a threshold for the NR2F6 staining intensity, we chose the mean staining intensity, as previously described. Additionally, we analyzed all results using the classification described by Klapper et al. (2020), constituting three groups according to the staining intensity. All our results presented here were qualitatively equal to our classification. We decided to use binary classification using the mean staining intensity to calculate statistical results providing adequate group sizes. The survival data showing a significantly better overall and progression-free survival in patients with NR2F6 overexpression suggest that the classification using the mean staining intensity might be adequate. Additionally, further statistical analyses are shown in the supplemental information.

Klepsch et al. showed that an inhibition of NR2F6 in T cells may increase the efficacy of PD-L1-targeting immune checkpoint therapies in mice (Klepsch et al. 2020). Hitherto, the precise pathways and influencing factors of T cells on carcinogenesis and cancer therapy are still mostly unknown. Furthermore, the chemosensitivity of NR2F6-positive carcinomas seems to be different to NR2F6-negative carcinomas (Zhang et al. 2020; Li et al. 2019). This may influence the therapy response and should be investigated.

Due to the retrospective nature of this study, the results need to be validated. Another limit of the study is the patient selection bias. All patients included in this analysis underwent a surgical treatment for endometrial cancer. Therefore, there are only a few patients with tumor stages III or IV according to the FIGO classification. This may explain the missing significance in the multivariate Cox regression analysis of the FIGO stages as an independent regression coefficient.

We conclude that NR2F6 might play a crucial role in endometrial cancer growth and cell differentiation. It might be independent from the known molecular subgroups. In this analysis, it shows an important impact on the overall and progression-free survival and, therefore, may serve as a prognostic marker. In the future, the impact of NR2F6 on chemosensitivity and on immune checkpoint therapy as well as the role of NR2F6 on effector T cells in endometrial carcinomas should be elucidated.

## Supplementary Information

Below is the link to the electronic supplementary material.

Supplementary file1 (DOCX 151 KB)

## Data Availability

Data are available upon request from the corresponding author.
