# Supplementary material for: Overexpression of the orphan nuclear receptor NR2F6 is associated with improved survival across molecular subgroups in endometrial cancer patients
Source: J Cancer Res Clin Oncol. 2023 Mar 8;149(10):7155–64. doi: 10.1007/s00432-023-04632-2 (PMC10374721; doi:10.1007/s00432-023-04632-2)
Supplement: Supplementary file 1 — Supplementary file1 (DOCX 151 KB) [file 432_2023_4632_MOESM1_ESM.docx]

**Overexpression of the Orphan Nuclear Receptor NR2F6 is associated with improved Survival across Molecular Subgroups in Endometrial Cancer Patients**

Proppe L^1*^, Jagomast T^2^, Beume S^1^, Klapper L.^2^ Gitas G^1,3^, Köster, F^1^, Perner S^2^, Rody A^1^, Ribbat-Idel J^2‡^, Hanker LC^1‡^

^1^Department of Gynecology and Obstetrics, University Medical Center Schleswig-Holstein, Campus-Lübeck, Lübeck, Germany ^2^Department of Pathology, University Medical Center Schleswig-Holstein, Campus-Lübeck, Lübeck, Germany ^3^Department of Gynecology and Obstetrics, University Medical Center Charité Berlin, Berlin, Germany

**Supplemental information**

To provide a as much data as possible we decided to add the Kaplan-Meier curves using the median NR2F6 antibody staining intensity in the supplemental information. All tumor samples with a staining intensity below or equal to the median staining intensity are classified to be NR2F6 negative. All samples with a staining above the median staining intensity are classified as NR2F6 positive.


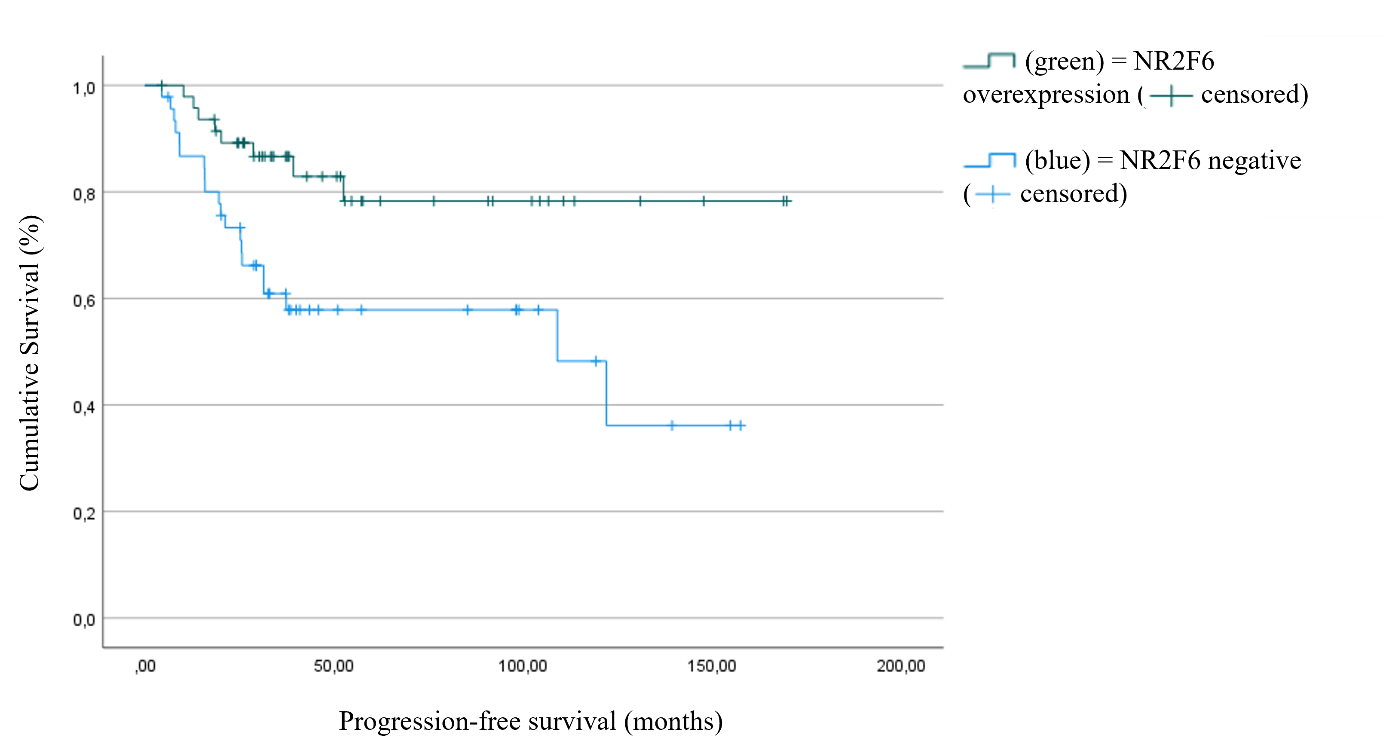


Fig S1. Progression-free survival (PFS) as a function of NR2F6 expression (*p* = 0.006, high NR2F6 expression *n*= 48, low NR2F6 expression *n* = 46).


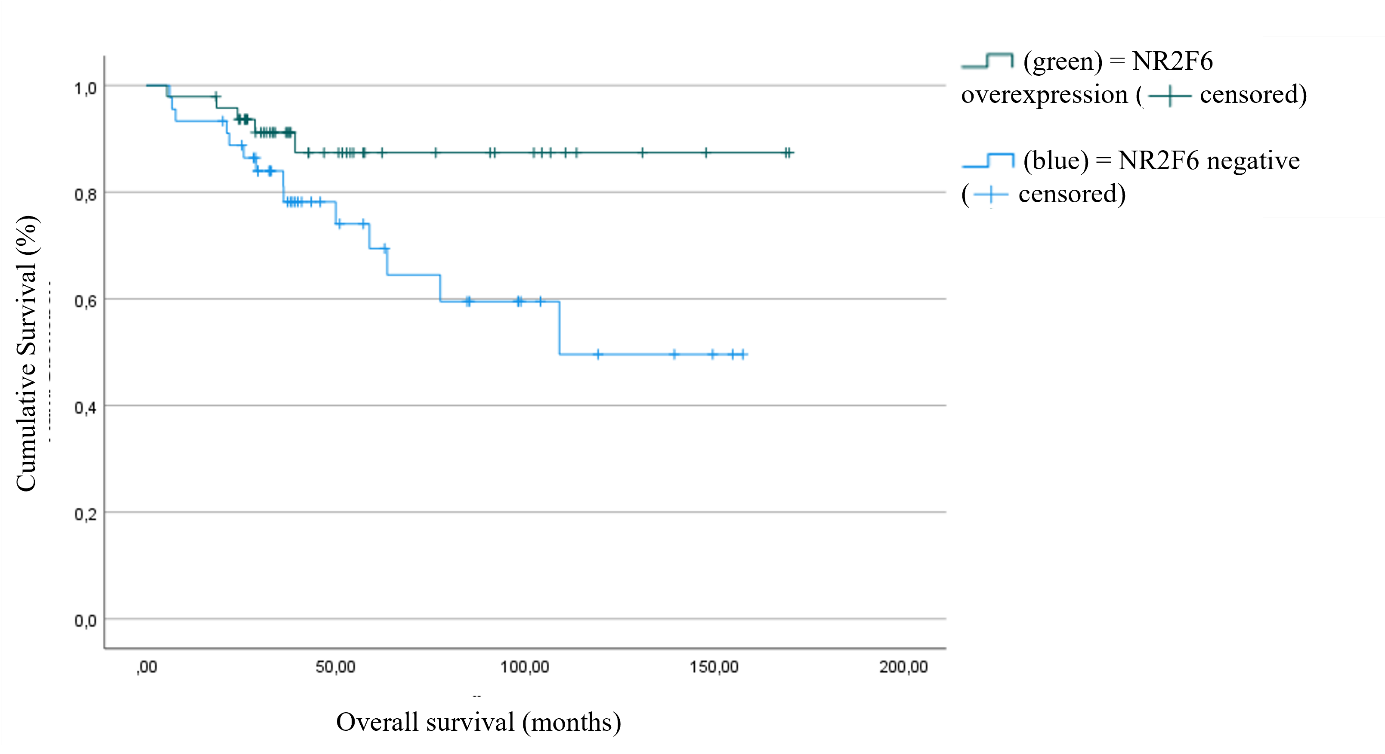


Fig S2. Overall survival (OS) as a function of NR2F6 expression (*p* = 0.03, high NR2F6 expression *n* = 48, low NR2F6 expression *n* = 45).
